# Measuring Appropriate Antibiotic Prescribing in Acute Hospitals: Development of a National Audit Tool Through a Delphi Consensus

**DOI:** 10.3390/antibiotics8020049

**Published:** 2019-04-29

**Authors:** Graeme Hood, Kieran S. Hand, Emma Cramp, Philip Howard, Susan Hopkins, Diane Ashiru-Oredope

**Affiliations:** 1 Public Health England, London SE1 8UG, UK; graeme.hood@nhs.net (G.H.); susan.hopkins@phe.gov.uk (S.H.); 2 University Hospital Southampton NHS Foundation Trust and School of Health Sciences, University of Southampton, Southampton SO16 6YD, UK; K.Hand@soton.ac.uk; 3 University Hospitals of Leicester NHS Trust, Leicester LE1 5WW, UK; emma.cramp@nhs.net; 4 Leeds Teaching Hospitals NHS Trust and University of Leeds, Leeds LS1 3EX, UK; Philip.howard2@nhs.net

**Keywords:** Antimicrobial resistance, antibiotics, antimicrobial stewardship, inappropriate prescribing, days of therapy, Start Smart then Focus

## Abstract

This study developed a patient-level audit tool to assess the appropriateness of antibiotic prescribing in acute National Health Service (NHS) hospitals in the UK. A modified Delphi process was used to evaluate variables identified from published literature that could be used to support an assessment of appropriateness of antibiotic use. At a national workshop, 22 infection experts reached a consensus to define appropriate prescribing and agree upon an initial draft audit tool. Following this, a national multidisciplinary panel of 19 infection experts, of whom only one was part of the workshop, was convened to evaluate and validate variables using questionnaires to confirm the relevance of each variable in assessing appropriate prescribing. The initial evidence synthesis of published literature identified 25 variables that could be used to support an assessment of appropriateness of antibiotic use. All the panel members reviewed the variables for the first round of the Delphi; the panel accepted 23 out of 25 variables. Following review by the project team, one of the two rejected variables was rephrased, and the second neutral variable was re-scored. The panel accepted both these variables in round two with a 68% response rate. Accepted variables were used to develop an audit tool to determine the extent of appropriateness of antibiotic prescribing at the individual patient level in acute NHS hospitals through infection expert consensus based on the results of a Delphi process.

## 1. Introduction 

Antimicrobial resistance (AMR) is a global threat to health affecting all healthcare systems and growing at an alarming pace [1,2]. In the UK an increase in annual secondary care antibiotic consumption by 4.8% (measured as defined daily doses (DDD) of antibiotics per 1000 inhabitants per day) was reported in England between 2013 to 2017 [3]. Optimising prescribing, through the development and implementation of antimicrobial stewardship (AMS) programmes is a key area of the UK five-year AMR strategy [4]. An important element of these programmes is assessing the quality of antimicrobial prescribing. Several countries now use European Centre for Disease Prevention and Control (ECDC) point prevalence survey (PPS) of healthcare-associated infections and antimicrobial use protocol to identify targets for quality improvement and evaluate antimicrobial stewardship programmes [5]. Surveillance data collected via PPS provide useful information for assessment of the burden of antimicrobial prescribing, however, the method has limited capacity to assess the appropriateness of prescribing in the absence of more complete individual patient-level clinical data [6]. An adaptation of the ECDC point prevalence survey tool was developed by infection experts in Australia to better measure prescribing quality; this tool performed well in validation, inter-rater reliability testing, and user feedback [7]. The Centers for Disease Control and Prevention (CDC) in the US have also published an audit tool to evaluate the quality of inpatient antibiotic prescribing with similar positive feedback [8]. These tools share common themes in their evaluation of appropriateness, such as use of individual patient clinical data and indication to aid the auditor’s judgement of appropriateness. Two important limitations of existing audit protocols remain evident: Firstly, application of a broad definition of inappropriateness that includes a range of prescribing quality indicators (such as prompt intravenous-to-oral switch and appropriate dosing) that do not predict overall consumption of antibiotics; and secondly, an inability to specifically quantify the scale of inappropriate prescribing directly resulting from unnecessary doses administered. An ability to quantify the proportion of antimicrobial consumption that is unnecessary will allow healthcare organisations and governments to determine targets for a reduction in antimicrobial consumption. This will limit avoidable selection pressure for AMR and can be achieved without compromising patient safety. In addition, whilst PPS can provide a rich data source due to the fact they require a point-in-time assessment, it is not possible to measure the total duration of antibiotic use for patients assessed as part of a PPS. The national audit tool designed for NHS hospitals described in this report aims to support a focussed patient-level assessment of the appropriateness of antibiotic prescribing in secondary care.

It is not currently possible to estimate with any certainty the proportion of antimicrobial prescribing in English hospitals that is inappropriate, as electronic prescribing data directly linking hospital prescriptions to clinical data are still not widely available. A key study aim was to develop and validate a patient-level audit tool that could support estimation of the number of days of antibiotic therapy that auditors considered non-essential and therefore potentially avoidable. The proportion of inappropriate antibiotic prescribing can be represented as non-essential days of therapy (DOTs) expressed as a percentage of total days of therapy. The goal of the audit tool was to collate variables that would support assessment of those aspects of prescribing most relevant for selection pressure and resistance. If successful, this would allow the UK to potentially set goals for reduction of antibiotic consumption in hospitals that are safe and achievable. A secondary aim included creating an audit tool that helped minimise subjectivity in order to standardise the assessment of appropriateness by prompting auditors to consider certain critical information in order to support their decision.

## 2. Results

### 2.1. Defining Appropriateness 

Defining the gold standard for appropriate antibiotic prescribing is challenging due to the inherently subjective nature of evaluating quality in prescribing. An initial list was compiled to incorporate elements of high-quality prescribing identified by opinion leaders in the field, the UK Government Scientific Advisory Committee on Antimicrobial Prescribing, Resistance and Healthcare Associated Infection (APRHAI) and a workshop including 22 infection experts convened to define appropriate antibiotic prescribing. The collated list (Table 1) sets out many elements of antibiotic prescribing that may be considered in an assessment of appropriateness. 

It could reasonably be argued that each of these elements is relevant to efficacy, safety, and improving patient outcomes, and critical to the assessment of appropriateness of prescribing. However, the APRHAI committee took a view that improvement in performance against certain elements would be less likely to impact upon the overall consumption of antibiotics and, consequently, would be less likely to impact upon antibiotic resistance. Three aspects were therefore given ultimate priority as most relevant to identifying avoidable selection of resistance within the UK hospital setting:

- Prescribing an antibiotic for a patient in the absence of (documented) evidence of bacterial infection.
- Prescribing a critical broad-spectrum antibiotic to patients in the absence of a (documented) rationale.
- Continuing an antibiotic prescription beyond the course length recommended in local or national guidelines, in the absence of a (documented) rationale.

### 2.2. Initial Draft of Audit Tool

A consensus emerged from the one-day workshop that existing antibiotic prescribing guidelines for acute hospitals often do not suggest clinical thresholds for initiating or stopping antibiotics. This means there is a lack of consistency between hospitals in assessing whether antibiotics are indicated and for how long. There was also general agreement that antibiotic consumption data alone were not sufficient to determine the proportion of prescribing that is inappropriate. This was further complicated by the failure of hospital-level antibiotic consumption data to account for patient acuity and hospital case mix. The workshop group arrived at the conclusion that an individual patient audit by an infection specialist was required to establish with greater certainty the current proportion of prescribing that is inappropriate. This process was anticipated to be less vulnerable to case mix or speciality bias. It was agreed that a patient-level audit tool would be sent for expert elicitation via a Delphi process. 

### 2.3. Round 1 Delphi

The response rate to the initial survey was 100% (19/19), taking on average 32 minutes to complete. Of the first-round responders, 42% (8/19) were specialist antimicrobial pharmacists, 21% (4/19) were infectious disease doctors or medical microbiologists, 16% (3/19) were general medical doctors, and the remaining 21% (4/19) were antimicrobial stewardship nurses. The majority (14/19) of participants in round one had over five years’ experience and practice in antimicrobial stewardship and infection. When asked what proportion of inpatients prescribed antibiotics should be audited to provide reasonable confidence of the overall assessment of the appropriateness of antibiotic prescribing in secondary care, 79% (15/19) expressed the view that either 5–10% or 10–25% of inpatients on antibiotics would be an adequate sample size to represent the entire hospital patient population. The panel members were also asked at what frequency would it be necessary and reasonable to repeat an audit assessing appropriate antibiotic prescribing; 89% (17/19) indicated annually and 11% (2/19) stated every three years would be sufficient. Twenty-three of the 25 variables were scored as relevant or highly relevant for assessing appropriateness for one or more of the main decision points (Supplementary Materials, Table S1) and were therefore accepted following Round 1. One variable, “documentation of pre-72-hour review”, was rejected by the expert panel in Round 1 as not relevant to the assessment of appropriateness at any of the main decision time points. A neutral consensus was reached by the expert panel for the variable “presenting complaint”; this was subsequently rescored in the second round. The first variable was renamed to “review of antibiotic prescription within 72 h” to remove the requirement for formal documentation of the review and presented for scoring in the second round. The project team was reluctant to remove this variable due to the potential value it brings in predicting unnecessarily prolonged treatment courses. 

### 2.4. Round 2 Delphi

**Figure 1:**
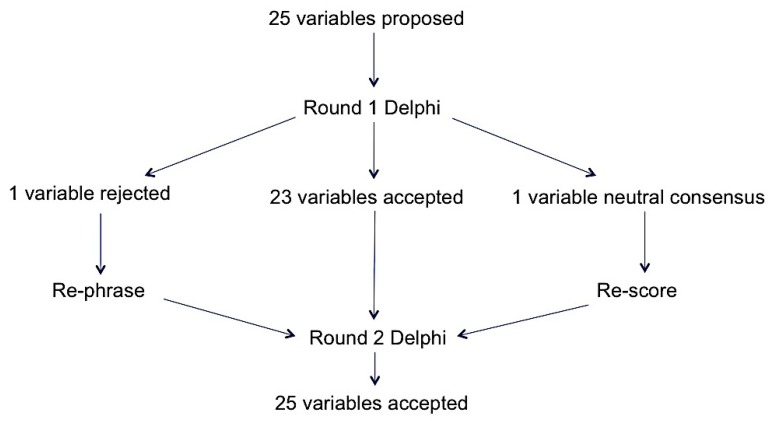
Overall Delphi results.

Out of the nineteen respondents (from Round 1) who were invited to participate in Round 2 the response rate was 68% (13/19). The same distribution of healthcare professionals participated in Round 2 with 69% (9/13) having over 5 years’ experience and practice in antimicrobial stewardship and infection. The neutral variable “presenting complaint” was rescored, with 85% (11/13) of respondents indicating this was considered relevant to assessing appropriate prescribing. A consensus was reached when the expert panel members were asked to score the renamed variable “review of antibiotic prescription within 72 h” with this being accepted by the panel (Figure 1).

The majority (9/13) of respondents reported that there were local guidance or supportive tools available to help clinicians stop antibiotic therapy safely. Where not available, three of the four respondents agreed that such guidance would be helpful. 

### 2.5. Feasibility

Eight of the 19 original panel members agreed that the audit tool was fit for purpose, 32% (6/19) expressed a neutral view, and 5 panel members disagreed (5/19) including one panel member stating they were unable to assess this question. The participants were asked if the time taken to complete the audit tool is a worthwhile investment of NHS resources for the benefit of patient safety and public health, with 43% (8/19) agreeing that it is, 21% (4/19) having a neutral view, 26% (5/19) disagreeing, and 10% (2/19) being unable to assess this.

## 3. Discussion

An audit tool consisting of 25 variables to support assessment of the appropriateness of antibiotic prescribing in secondary care has been developed. The prototype audit tool was reviewed by 19 multi-disciplinary infection specialist health professionals through a two stage modified Delphi process and all 25 variables were accepted by specialists as relevant or very relevant to the aim of the audit following rewording of just one variable.

The response rate of participants within the Delphi process was seen to reduce between rounds one and two. A systematic review of 31 studies indicated that the median response rate of a Delphi process does reduce by 2% from the first to the last round of Delphi processes [30], however even with reminder e-mails our response reduced by 32% from round one to two, so this could be interpreted as introducing potential bias. The Delphi process occurred during winter months which can operationally be a busy time for NHS hospitals. This could have been a contributing factor in the reduced response rate seen over the two rounds. 

The inclusion of acuity scores within the audit tool was to help support the auditor in assessing appropriateness of antibiotics by highlighting the severity of the acute health status which could be as a result of a likely infection. The supportive use is important as a low acuity score does not rule out infection, but merely indicates no severe or deteriorating condition or a lesser probability of organ failure. Evidence from a multi-centre study of adult emergency departments in the UK reported a positive predictive value for National Early Warning Score (NEWS) ≥3 of 20% for mortality or ICU admission [31]. It was therefore considered reasonable to assess what proportion of patients treated with antibiotics either have NEWS ≥3 or localised evidence of infection at an anatomical site. An abbreviated version of the Sequential Organ Failure Assessment (SOFA) score, quick SOFA (qSOFA), was assessed in almost 75,000 adult patients hospitalised with suspected infection [32]. The predictive validity of the qSOFA score for in-hospital mortality was found to be statistically greater than the SOFA or systemic inflammatory response syndrome (SIRS) scores, supporting its use as a prompt to consider possible sepsis [32]. Applying qSOFA score ≥2 during emergency department or ward stay as a prediction tool for in-hospital all-cause mortality had a negative predictive value of 97% in adult patients with clinical suspicion of infection in a recent multi-centre study including 27 French emergency departments [33]. A qSOFA score at a threshold of 2 or more may be considered by some clinicians to be a reasonable tool for differentiating patients in whom immediate broad-spectrum antibiotics are justified, given the associated in-hospital mortality of 24% [33]. 

During the second round of the Delphi process the panel did not accept that formal documentation of a pre-72-hour review was relevant. The panel did however accept that whether a pre-72-hour review took place was relevant, irrespective of documentation (e.g., prescription changed but no corresponding entry in the medical notes) for the purposes of assessing appropriateness of antibiotic prescribing. In the absence of a pre-72-hour review, it is likely that patients for whom diagnostic tests support an alternative diagnosis to infection are likely to continue antibiotics longer than necessary. This variable is also important due to the potential value it brings to predicting unnecessarily prolonged treatment courses, and it is part of ongoing quality improvement work within English hospitals, so the indicator was reworded for Round 2. Secondary healthcare quality improvement work within England has focussed on reducing total antibiotic consumption as well as obtaining evidence of antibiotic review within 72 h of commencing an antibiotic [34]. 

The addition of separate questions to explore individual views on inappropriate prescribing within secondary care was used as it was not possible to estimate a sample size, because the proportion of non-essential DOTs was unknown. Delphi participants were asked what proportion of patients on antibiotics required auditing to give them assurance on appropriateness of antibiotic prescribing for their hospital. 

The expert panel members were invited to estimate the feasibility and likely value of the audit tool; this saw 26% of the expert panel disagreeing with statements that the audit tool was fit for purpose or a worthwhile investment of NHS resources. This finding was surprising given the high degree of consensus over the relevance of individual variables for supporting the assessment of appropriateness and was not explored in more detail. It may reflect a perception of futility that a simple audit tool could not sufficiently capture the complexity of an episode of infection to enable a reproducible assessment of appropriateness. A pilot study of the audit tool to test the reliability of the instrument is clearly a prerequisite before more widespread adoption.

### 3.1. Study Strengths and Limitations

Strengths of this project include the development of an audit tool through a consensus of a multi-disciplinary team of experienced infection specialists working in hospital clinical practice. The authors believe the developed audit tool is the first of its kind internationally that can specifically quantify the scale of inappropriate prescribing directly resulting from unnecessary doses being administered. The audit tool was designed to minimise the burden of data collection by limiting the items of data collected to only those considered relevant or highly relevant to the specific aim of evaluation of appropriateness of prescribing at three critical prescribing decision time points. The audit tool also narrows the focus of the audit to evaluate only those elements of prescribing associated with unnecessary antibiotic doses and therefore likely to be most relevant to the selection of resistant microorganisms: whether an antibiotic is indicated at the start of treatment, whether treatment beyond 72 h is justified, and whether treatment beyond a standard course length is appropriate. 

The potential to collect more detailed clinical information to explore the subjective decision that the auditors would take was advantageous. Standardisation of the assessment process may be aided by the inclusion of objective variables to capture evidence of infection or sepsis, along with markers of severity including qSOFA score, NEWS, C-reactive protein (CRP), and white blood cell count. 

The potential limitations of the audit tool include the time needed to collect data and risk of subjectivity in assessment which are inherent limitations of this approach, however a balance is struck between the value of detailed patient-level data to robustly answer the important question of appropriateness set against the use of a standardised data collection tool to minimise variability in assessment. There was also limited opportunity for experts to consider variables not included in the tool, such as medical imaging and urine dipstick tests. Presenting an increased number of potentially relevant variables such as antibiotic dose and route of administration may have provided greater opportunity to discriminate between the perceived value of different variables for estimating non-essential doses. Whilst a relatively small sample of experts participated in the Delphi process, an important limitation, it is worth noting that 22 experts participated in the workshop to define appropriate antibiotic prescribing and develop the initial audit tool cascaded for the Delphi consensus. Subsequent piloting of the audit tool will need to include ongoing evaluation of the strengths and weakness of the tool and inform iterative improvements. The minor changes to the audit tool as a result of this Delphi process provide reassurance that extensive revision of the tool was not required, however a higher number of experts participating would have provided greater assurance of a reliable rating for each of the variables assessed. 

Since admission and discharge dates were not included in the tool it was not possible to determine at which point during their admission a patient receives antibiotics and therefore whether a patient is receiving treatment for a healthcare-associated infection or community-acquired infection unless this is stated explicitly by the auditor. This was identified by the project team as a priority to capture in future versions of the audit tool. 

The use of a web-based Delphi process limited opportunity for qualitative feedback from a face-to-face discussion and may have contributed to the lower response rate for Round 2. The selection of expert panel members was via national infection networks and only one healthcare professional also participated in the previous workshop that convened to define appropriate prescribing. To help reduce potential bias, all participants were blinded to who participated in the expert panel with individual correspondence being sent. 

The use of the developed audit tool is for active treatment and does not assess long-term and peri-operative surgical prophylaxis antibiotics. An adaptation could be made to the audit tool in order to assess these groups of patients. Finally, the developed audit tool was prioritised for antibacterial agents only but could also be adapted for antifungal and antiviral agents. 

### 3.2. Future Work

Piloting the audit tool within NHS hospitals to assess usability and operability is the next step. This would also provide additional insight and development of the tool and mitigate against the limitations of the small consensus panel. The resource burden of data collection and whether there is variation between hospitals that use electronic systems or paper-based processes needs to be quantified. Although it is anticipated that the use of electronic systems should be faster than paper-based prescribing, ease of access to information for each variable may vary. Piloting the audit tool within 5 to 10 hospitals could provide a preliminary assessment of non-essential days of antibiotic therapy prescribed to inform the sample size calculation for a definitive study. Recruiting a mixture of hospital types (teaching, district and specialist) will be important to assess the practicality of the developed audit tool. The balance between the amount of information being collected with the time taken to collect the data in a busy operational hospital will be crucial. 

## 4. Materials and Methods 

### 4.1. Part 1: Development of the Audit Tool

Evidence synthesis of published literature and international guidance contributed to the development of the initial prototype audit tool to try and quantify and identify inappropriate antimicrobial prescribing. The initial prototype audit tool then underwent preliminary feasibility testing at one acute teaching hospital in Southampton, England. This was conducted by a specialist pharmacist supported by a medical microbiologist, with a focus on evaluating the operability of the audit tool. After a subgroup of the UK government APRHAI scientific advisory committee drafted standards to define appropriate prescribing in secondary care a finalised prototype audit tool was created [4]. The subgroup discussed aspects of auditing including frequency, intensity, resourcing, and mechanism of data collection including feedback and reporting processes. 

To elicit further expert opinion, a one-day workshop was held on 6 March 2017 with infection experts including secondary care clinicians (10/22), pharmacists (9/22), a nurse, an academic, and a medical director to explore views on the appropriateness of prescribing within hospitals. The project team including two pharmacists of the antimicrobial resistance programme at Public Health England and three national antimicrobial pharmacists met to discuss the workshop findings and updated the audit tool accordingly. Elements of the audit tool that were intended to support the assessment of appropriateness of antimicrobial prescribing were termed “variables” for the purposes of the validation process. The selection of variables to include in the audit tool was based on discussions within the antibiotic prescribing quality subgroup of APRHAI, iterative e-mail feedback from co-authors, and prototype audit tool testing in Southampton.

To support the auditor in their assessment of the severity of infection and deterioration as a result of a likely infection the National Early Warning Score (NEWS) and quick sequential organ failure assessment (qSOFA) score were incorporated into the audit tool [32,35]. A threshold NEWS value of 3 or above has been proposed as a suitable trigger to systematically screen for sepsis or septic shock in patients with suspected infection in a United States Emergency Department (ED) population with a reported negative predictive value of 99.5 (95% CI 97.8–99.9) [36]. 

### 4.2. Part 2: Validation of the Audit Tool

Between October and December 2017, a RAND-modified Delphi process was conducted to validate each individual variable within the audit tool. A multi-disciplinary expert panel of infection and public health specialists was recruited via e-mail through national infection and microbiology networks and asked to assess the relevance of each variable against a five-point Likert Scale [37]. The relevance was assessed in relation to three main decision time points during a course of antibiotic therapy:

- Initiation (was the antibiotic indicated and necessary at the start date?);
- Early post prescription review (was the antibiotic continued after infection was ruled out?);
- End of therapy (was the antibiotic continued beyond the standard duration?).

Non-essential days of antibiotic therapy were included with potential reasons as follows: (1) antibiotic not indicated/unnecessary at start date; (2) unexplained continuation of antibiotic after infection ruled out; and (3) unexplained continuation of antibiotic beyond standard duration. The standard duration was defined by individual hospitals as identified in local antibiotic guidelines. A separate questionnaire was sent to the Delphi participants to explore individual views on inappropriate prescribing within secondary care. 

The expert panel participated in two rounds. Invitation via e-mail was sent to 19 participants before the first round. The online survey SelectSurvey.net was used in each Delphi round with participant reminder e-mails sent periodically. The results were discussed within the project team after the first round and the aggregated scores were shared with the participants prior to the second round. Each of the 25 variables was assessed for its relevance against the three main decision time points. Qualitative feedback was reviewed by one project team member and common themes reported for discussion with the full project team. The threshold for accepting a variable was met if the median score was “relevant” or “very relevant” for assessing appropriateness at any one of the three decision time points. A variable was rejected if the median score for all three of the critical decision points was “irrelevant” or “very irrelevant” with a neutral score being rescored in the second round to provide a definitive response. The rewording of questions between each round occurred by the project team and was presented to the participants from Round 1 for Round 2. After completing both rounds of the Delphi process the accepted variables were incorporated into a finalised audit tool (Supplementary Materials, Figure S1).

### 4.3. Statistics

Data were summarised using non-parametric descriptive statistics which are considered suitable to measure consensus and stability in Delphi studies [38] and due to the number of Delphi participants. The median and percentage “relevant” and “highly relevant” responses for each of the variables in relation to each decision time point was calculated using univariate analysis.

## 5. Conclusions

In summary, the RAND-modified Delphi process has provided face validity of this audit tool and selection of critical variables that can be used to support the assessment of inappropriate antibiotic prescribing. The feedback from expert panel members helped shape development of the audit tool and provides an important endorsement from practising healthcare professionals within and outside the NHS. This audit methodology will help hospitals identify the location, extent, and nature of inappropriate antibiotic prescribing and provide evidence to drive local antimicrobial stewardship and focus targeted training to specific areas of improvement.

## Figures and Tables

**Table 1:** Elements of antibiotic prescribing in hospitals relevant for evaluating appropriateness [9,10,11,12,13,14,15,16,17,18,19,20,21,22,23,24,25,26,27,28,29].

Prescribing Elements (Potential Audit Variables)	Comments	Selected for Audit
**START SMART**		
No antibiotic if not indicated (no reasonable evidence of infection)	Unnecessary antibiotic exposure selects for avoidable resistance [9,10,11].	✓
Indication documented	Good practice for continuity of care but of uncertain relevance to resistance.	✓
Appropriate specimens taken for microscopy, culture, and sensitivity (MC&S)—blood cultures and suspected site of infection	Important for establishing evidence of infection and for targeting appropriate therapy but requires manual audit and >50% of cultures are negative [12,13].	✓
No allergy or contra-indication to treatments	Important patient safety consideration but not relevant for resistance.	✕
Prompt administration of first dose	Important patient safety consideration in cases of severe sepsis but of uncertain relevance to resistance. Already captured by national sepsis audits.	✕
Treatment regimen adequate to cover most likely pathogens	Meta-analysis of RCTs reports increased risk of mortality if initial regimen inadequate [14]. Relevance to resistance uncertain.	✓ *
Treatment regimen not unnecessarily broad spectrum	Indiscriminate use of critical broad-spectrum agents unnecessarily selects for resistance [15,16,17].	✓ *
No redundant agents in treatment regimen	Unnecessary antibiotic exposure selects for avoidable resistance [9,10,11].	✓
Treatment regimen compliant with local/national guideline or justified deviation	Validity dependent upon quality of local guideline. Relevance to resistance uncertain.	✕
Treatment regimen cost-effective	Not relevant to resistance.	✕
No underdosing	Limited evidence from modeling suggests that low doses may select resistance in pneumococci [18] but underdosing unlikely to be a problem in NHS hospitals due to pharmacist and nurse intervention.	✕
No overdosing	Important patient safety consideration but likely to reduce rather than increase risk of selecting resistance [19,20,21,22,23,24].	✕
Correct route of administration	Relevant for efficacy, length of stay, and risk of line infection but of uncertain relevance to resistance.	✕
Prompt appropriate source control	Subjective assessment. Of uncertain relevance to resistance.	✕
No missed doses or delayed doses	Of uncertain relevance to selection of resistance.	✕
Therapeutic drug monitoring (TDM) for narrow therapeutic index drugs	Important primarily for patient safety (but also for efficacy); of uncertain relevance to resistance.	✕
**THEN FOCUS**		
Prompt discontinuation of antibiotics if alternative diagnosis established and infection excluded	There is RCT evidence that unnecessary continuation selects for multi-resistant organisms [25,26,27].	✓
Appropriate broadening of spectrum in response to MC&S results	This may necessitate an increase in broad-spectrum agent use if indicated by MC&S results. Failure to adjust ineffective treatment to MC&S results is associated with a higher risk of mortality [27].	✓ *
Appropriate narrowing of spectrum in response to MC&S results	Evidence largely from observational studies suggests that de-escalation to narrow-spectrum agents is safe when patients are improving clinically and a plausible pathogen has been identified [28].	✓ *
Prompt referral to outpatient parenteral antibiotic therapy OPAT services for suitable patients	Relevant for length of stay and risk of healthcare-associated infection (HCAI) but of uncertain relevance to resistance.	✕
Prompt switch from IV to oral route of administration when safe and effective	Relevant for length of stay and risk of line infection but of uncertain relevance to resistance.	✕
Antibiotic plan documented in the notes	Good practice for continuity of care but of uncertain relevance to resistance.	✕
No unjustified prolonged duration of treatment	There is evidence from RCTs and observational studies that unnecessarily prolonged duration selects for multi-resistant organisms [25,26,29]. Can only be audited at the end of therapy.	✓

* Prescribing elements relating to antibiotic spectrum; deprioritised for audit tool prototype

## Supplementary Materials

The following are available online at https://www.mdpi.com/2079-6382/8/2/49/s1. Figure S1: Assessing appropriateness of antibiotic therapy–final national audit tool for pilot. Table S1: Round 1 Delphi results.
