# Supplementary material for: Measuring Appropriate Antibiotic Prescribing in Acute Hospitals: Development of a National Audit Tool Through a Delphi Consensus
Source: Antibiotics (Basel). 2019 Apr 29;8(2):49. doi: 10.3390/antibiotics8020049 (PMC6627925; doi:10.3390/antibiotics8020049)
Supplement: Supplementary file 1 [file antibiotics-08-00049-s001.zip › antibiotics-490220-supp -.pdf]

Table S1: Round 1 Delphi Results

Variable	Was the antibiotic indicated and necessary at the start date?		Was the antibiotic continued after infection was ruled out?		Was the antibiotic continued beyond the standard duration without explanation?		Round 1 Decision
	**Median Score**	**Relevant + Very Relevant Response (% of participants)**	**Median Score**	**Relevant + Very Relevant Response (% of participants)**	**Median Score**	**Relevant + Very Relevant Response (% of participants)**	
1. Working diagnosis	Very Relevant	90%	Relevant	68%	Relevant	58%	Accepted
2. Presenting complaint	Neutral	53%	Neutral	32%	Irrelevant	32%	Rescore
3. Documentation of pre-72 hour review	Relevant/ Neutral	56% (1 respondent could not assess)	Relevant/ Neutral	47% (1 respondent could not assess)	Neutral	47% (2 respondents could not assess)	Rescore
4. Documentation of working diagnosis	Relevant	74%	Relevant	53%	Neutral	53%	Accepted
5. Patient immunocompromised	Relevant	74%	Neutral	42%	Relevant	53%	Accepted
6. Evidence	Relevant	79%	Neutral	37%	Neutral	47%	Accepted

of local infection							
7. Confirmation of infection at pre-72 hour review	Very Relevant	74%	Relevant/ Neutral	50% (1 respondent could not assess)	Relevant	58%	Accepted
8. Standard duration of treatment for indication	Neutral	53%	Irrelevant	21%	Very Relevant	90%	Accepted
9. Finalised diagnosis	Very Relevant	63%	Neutral	63%	Relevant/ Neutral	68%	Accepted
10. NEWS score at course start	Relevant	84%	Neutral	32%	Neutral	42%	Accepted
11. NEWS score at day 5-7	Irrelevant	21%	Neutral	42%	Relevant	53%	Accepted
12. qSOFA score at course start	Relevant	67% (1 respondent could not assess)	Neutral/ Irrelevant	6% (1 respondent could not assess)	Neutral	16% (1 respondent could not assess)	Accepted
13. qSOFA score at day 5-7	Irrelevant	13% (3 respondents could not assess)	Neutral	29% (2 respondents could not assess)	Relevant	53% (2 respondents could not assess)	Accepted
14. Highest CRP level in previous 24 hours at course start	Relevant	84%	Neutral	32%	Neutral	37%	Accepted

15. Highest CRP level in previous 24 hours at day 5-7	Irrelevant	21%	Relevant	47%	Relevant	63%	Accepted
16. Highest WBC level in previous 24 hours at course start	Relevant	79%	Neutral	42%	Relevant	47%	Accepted
17. Highest WBC level in previous 24 hours at day 5-7	Irrelevant	26%	Neutral	42%	Relevant	63%	Accepted
18. Drug name	Very Relevant	58%	Neutral	47%	Relevant	53%	Accepted
19. Start date and time of drug	Relevant	63%	Neutral	47%	Relevant	53%	Accepted
20. Stop date and time of drug	Irrelevant	32%	Relevant	63%	Relevant	68%	Accepted
21. Antibiotic indicated	Very Relevant	82% (2 respondents could not assess)	Relevant	59% (2 respondents could not assess)	Relevant	47% (2 respondents could not assess)	Accepted
22. Blood culture identified	Relevant	63%	Relevant	74%	Relevant	74%	Accepted

23. Other specimens sent	Neutral	37%	Relevant	63%	Relevant	53%	Accepte d
24. Antibiotic necessary in regimen	Very Relevan t	75% (3 respondents could not assess)	Neutral	44% (3 respondents could not assess)	Relevant	50% (3 respondents could not assess)	Accepte d
25. Pathogen identified	Relevan t	68%	Relevant	63%	Very Relevant	79%	Accepte d
