# Supplementary material for: Measuring Appropriate Antibiotic Prescribing in Acute Hospitals: Development of a National Audit Tool Through a Delphi Consensus
Source: Antibiotics (Basel). 2019 Apr 29;8(2):49. doi: 10.3390/antibiotics8020049 (PMC6627925; doi:10.3390/antibiotics8020049)
Supplement: Supplementary file 1 [file antibiotics-08-00049-s001.zip › antibiotics-490220-supp-.pdf]

ANTIBIOTIC APPROPRIATENESS – END OF COURSE ASSESSMENT – DRAFT v5.0

Audit to be performed for a single episode of infection in hospitalised ADULTS at the end of a course of therapy (or if stop-date confirmed)

*Data for submission to PHE ?=Unknown

AUDIT ADMIN			
Audit date:		Audit number*:	
Auditor name:		Hospital number:	
Auditor profession:	Infection specialist?* Y / N	Age & gender:	yrs M / F
		Ward:	
Total data collection time:	minutes *	Specialty*:	

MICROBIOLOGY		
Blood culture?	Y / N	Auditor notes:
Other specimens sent?	Y / N	
Pathogen identified?	Y / N	

MEDICAL NOTES	
Presenting complaint:	
Working infection diagnosis:	
Patient immunocompromised?§	Y / N *
Evidence of local infection at anatomical site?	Y / N
Details of local infection:	
Review of antibiotic prescription within 72h?	Y / N / ? *
Finalised infection diagnosis*:	
Infection confirmed at pre-72 hour review and ongoing antibiotic therapy indicated?	Y / N/ ? *
Standard duration of treatment for indication:	days *
Explanation for prolonged treatment:	

OBSERVATIONS / VITAL SIGNS	Course start	Day 5-7
Highest **NEWS** result in previous 24h before abx:<br>(See overleaf for NEWS table)	Value =	Value =
	***NEWS ≥3:**	
	Y / N / ?	Y / N / ?
Highest **qSOFA** score in previous 24h before abx:<br>Score 1 point each for:<br>• Respiratory rate >21/min<br>• Blood pressure systolic <100 mmHg<br>• Altered mental status	Course start	Day 5-7
	Value =	Value =
	***qSOFA ≥2:**	
	Y / N / ?	Y / N / ?
LABORATORY RESULTS	Course start	Day 5-7
Highest C-Reactive Protein (CRP) level in previous 24-48h (normal range <10mg/L):	mg/L	mg/L
Highest or lowest white blood cell (WBC) count in previous 24-48h (normal range 4 – 11 × 10^9/L):	× 10^9/L	× 10^9/L

DRUG CHART / PRESCRIPTION					APPROPRIATENESS (AUDITOR OPINION)		**Record any days of non-essential therapy for <u>one</u> of 3 reasons**		
	**Drug name**	**Start date / time**	**Stop date / time**	**Days of therapy**	**Antibiotic indicated** * (evidence consistent with local infection or sepsis)	**AND antibiotic necessary**¥ **in regimen** *	1. Antibiotic not indicated/ unnecessary at start date	2. Unexplained continuation after infection ruled out	3. Unexplained continuation beyond standard duration
**Antibiotic 1**				days	Y / N	Y / N	days	days	days
**Antibiotic 2**				days	Y / N	Y / N	days	days	days
**Antibiotic 3**				days	Y / N	Y / N	days	days	days
**Antibiotic 4**				days	Y / N	Y / N	days	days	days
**Antibiotic 5**				days	Y / N	Y / N	days	days	days
**Total days of therapy (all antibiotics)**				days*	Total days of non-essential antibiotic therapy		days*	days*	days*
Course length (earliest start date to last stop date)				days	**GRAND TOTAL days of non-essential antibiotic therapy (sum of 1 + 2 + 3)**		days*		

¥ Definition of necessary = agent typically active against expected pathogens and no redundant overlapping spectrum with another agent in the regimen.

National Early Warning Score (NEWS)								
**PHYSIOLOGICAL PARAMETERS**	**3**	**2**	**1**	**0**	**1**	**2**	**3**	**Score**
**Respiration Rate**	≤8		9-11	12-20		21-24	≥25	
**Oxygen Saturations**	≤91	92-93	94-95	≥96				
**Any Supplemental Oxygen**		Yes		No				
**Temperature**	≤35.0		35.1-36.0	36.1-38.0	38.1-39.0	≥39.1		
**BP Systolic**	≤90	91-100	101-110	111-219			≥220	
**Heart Rate**	≤40		41-50	51-90	91-110	111-130	≥131	
**Level of Consciousness**				A			V, P or U	
**GRAND TOTAL SCORE**							**TOTAL:**	

AVPU = Alert; responds to Voice; responds to Pain; Unresponsive
Royal College of Physicians, Royal College of Nursing, National Outreach Forum and NHS Training for Innovation, July 2012

EXPLANATORY NOTES FOR AUDITORS

Audit of Appropriateness of Antibiotic Therapy for a Single Episode of infection in Adult Patients in an Acute Hospital Setting – DRAFT v5.0

Aim

This audit aims to estimate what proportion of antibiotic (antibacterial) days of therapy are non-essential in the judgement of the auditor and therefore potentially avoidable.

Rationale

The focus on avoidable days of therapy is deliberate; to establish whether there is room for improvement and to identify improvement goals that are safe for patients and relevant for antibiotic resistance. Assessment of appropriateness is subjective but hospitals that report minimal room for improvement in comparison to their peers may be subject to validation audit. Auditors may wish to document a brief narrative record of why some treatment days were judged to be "non-essential". This audit tool is not designed to capture information on off-guideline prescribing or overuse of broad-spectrum or IV antibiotics.

Sampling

Include adult patients at the end or nearing the end of a course of treatment for a single episode of infection (completing treatment courses on the ward or on discharge prescriptions). Aim to sample randomly across a range of medical and surgical specialties. *The audit is not designed to evaluate peri-operative surgical prophylaxis.*

Data for submission to Public Health England (PHE)

Data items for submission to PHE are indicated with an asterisk. This is a minimum dataset to reduce workload associated with this audit. The majority of data items included in this audit tool are for the benefit of the auditor when estimating appropriateness and to allow the auditor to maintain a record of the source patient and date. A template Microsoft Excel spreadsheet will be provided to faciliate recording of all data by Trusts that wish to do so on a voluntary basis.

§Definition of immunocompromised

Consistent with the "Green Book". i.e. Any of: Immunodeficiency syndrome; HIV infection; Bone marrow or stem cell transplant; Chemo / radiotherapy within 6 months; High-dose steroids >/=40mg prednisolone/day for >7days; or Immunosuppressant drugs.

NEWS and qSOFA

For patients with no apparent evidence of local infection at an anatomical site, the NEWS and qSOFA scores are provided as an aid for auditors to identifying sepsis of uncertain origin and also to capture apparent discordance between clinical findings and prescribing behaviour. *These scores have not been validated in paediatric patients.*

Applying NEWS ≥3 as a screening threshold for severe sepsis (Surviving Sepsis Campaign 2012 criteria) had sensitivity of 93% and a negative predictive value of 99.5% in an Emergency Department (ED) setting in London [Keep JW et al, 2016]. A study of over 27,000 adult patients admitted to 20 Scottish hospitals identified almost 20% meeting the Surviving Sepsis Campaign 2012 criteria for sepsis; only 9.7% of these patients had a NEWS <3 before leaving the ED [Corfield AR et al, 2014].

The qSOFA score is advocated as a tool for predicting in-hospital mortality in patients with suspected infection, supporting its use as a prompt to consider possible sepsis [Seymour CW et al, 2016]. The qSOFA is not an alert that alone will differentiate patients with infection from those without infection. Applying qSOFA score ≥2 during ED stay as a prediction tool for in-hospital all-cause mortality had a negative predictive value of 97% in patients with clinical suspicion of infection in a recent multi-centre study including 27 French EDs [Freund Y et al, 2017]. Patients with a qSOFA score of 2 or higher had an in-hospital mortality rate of 24% compared with 3% for patients with a qSOFA score of less than 2. Both NEWS ≥3 and qSOFA score ≥2 have negative predictive values for in-hospital mortality of 97% in adult hospitalised patients outside the ICU with suspected infection [Churpek MM et al, 2017].
